# A review of anti-tumour effects of *Ganoderma lucidum* in gastrointestinal cancer

**DOI:** 10.1186/s13020-023-00811-y

**Published:** 2023-08-28

**Authors:** Ting Ye, Yang Ge, Xiaoying Jiang, Hang Song, Can Peng, Bin Liu

**Affiliations:** 1 School of Integrated Chinese and Western Medicine, Anhui University of Chinese Medicine, Hefei, 230012 China; 2 Department of Technology, Beijing Chest Hospital, Capital Medical University/Beijing Tuberculosis and Thoracic Tumor Research Institute, Beijing, 101149 China; 3 Anhui Province Key Laboratory of Translational Cancer Research, Bengbu Medical College, Bengbu, 233030 China; 4 School of Pharmacy, Anhui University of Chinese Medicine, Hefei, 230012 China; 5 Cancer Research Centre, Beijing Chest Hospital, Capital Medical University/Beijing Tuberculosis and Thoracic Tumor Research Institute, Beijing, 101149 China

**Keywords:** *G. lucidum*, GI cancer, Ganoderic acid, Molecular mechanism, Clinical application

## Abstract

Gastrointestinal (GI) cancer is the most common cancer in the world and one of the main causes of cancer-related death. Clinically, surgical excision and chemotherapy are the main treatment methods for GI cancer, which is unfortunately accompanied with serious adverse reactions and drug toxicity, bringing irreversible damage to patients and seriously affecting the quality of life. *Ganoderma lucidum* (*G. lucidum*) has a long history of medicinal and edible use in China. Its bioactive compounds mainly include polysaccharides, triterpenes, and proteins, which have potential anti-tumor activities by inhibiting proliferation, inducing apoptosis, inhibiting metastasis, and regulating autophagy. Currently, there is no in-depth review on the anti-tumor effect of *G. lucidum* in GI cancer. Therefore, this review is an attempt to compile the basic characteristics, anti-GI caner mechanisms, and clinical application of *G. lucidum*, aiming to provide a reference for further research on the role of *G. lucidum* in the prevention and treatment of GI cancer from the perspective of traditional Chinese and western medicine.

**Graphical Abstract:**

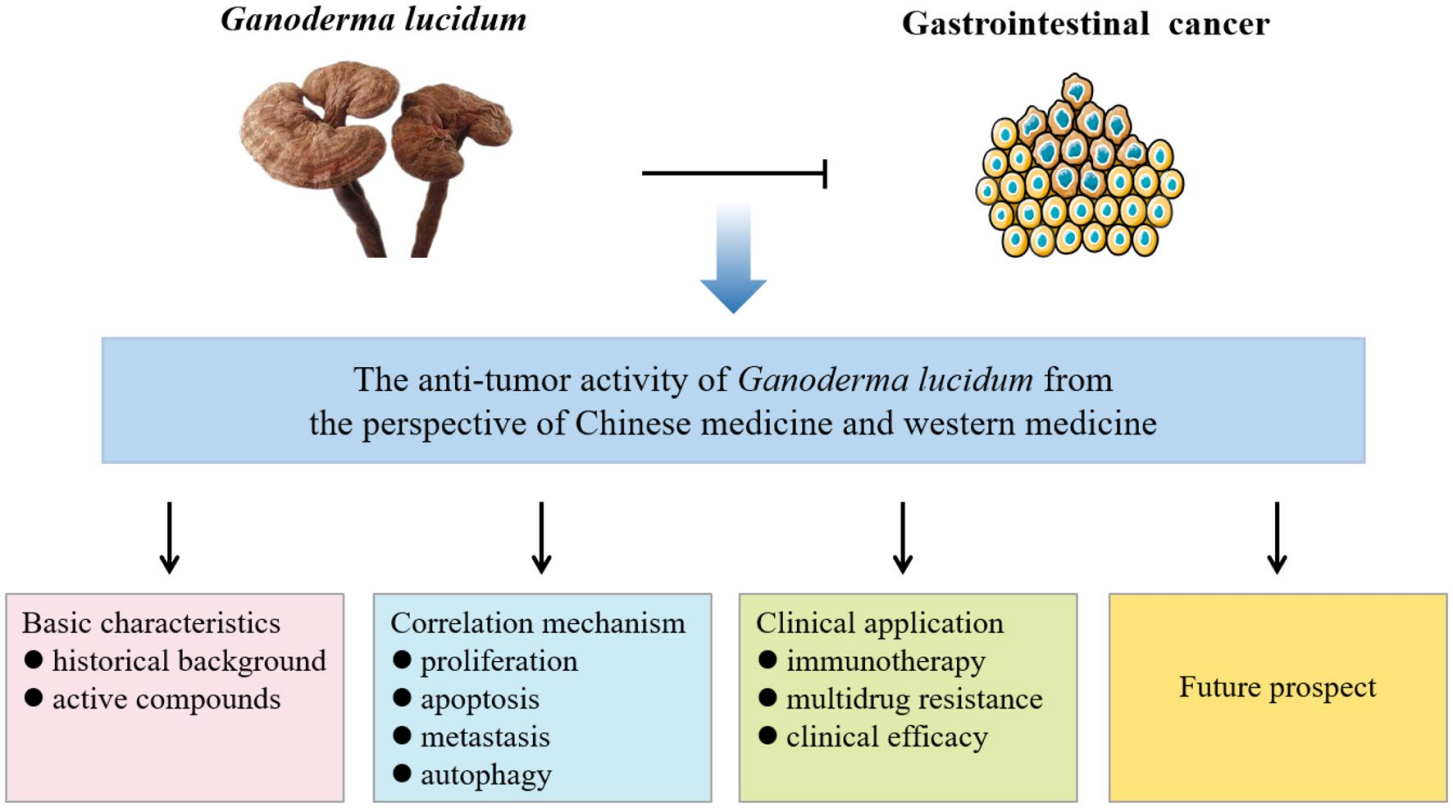

## Introduction

**Fig. 1:**
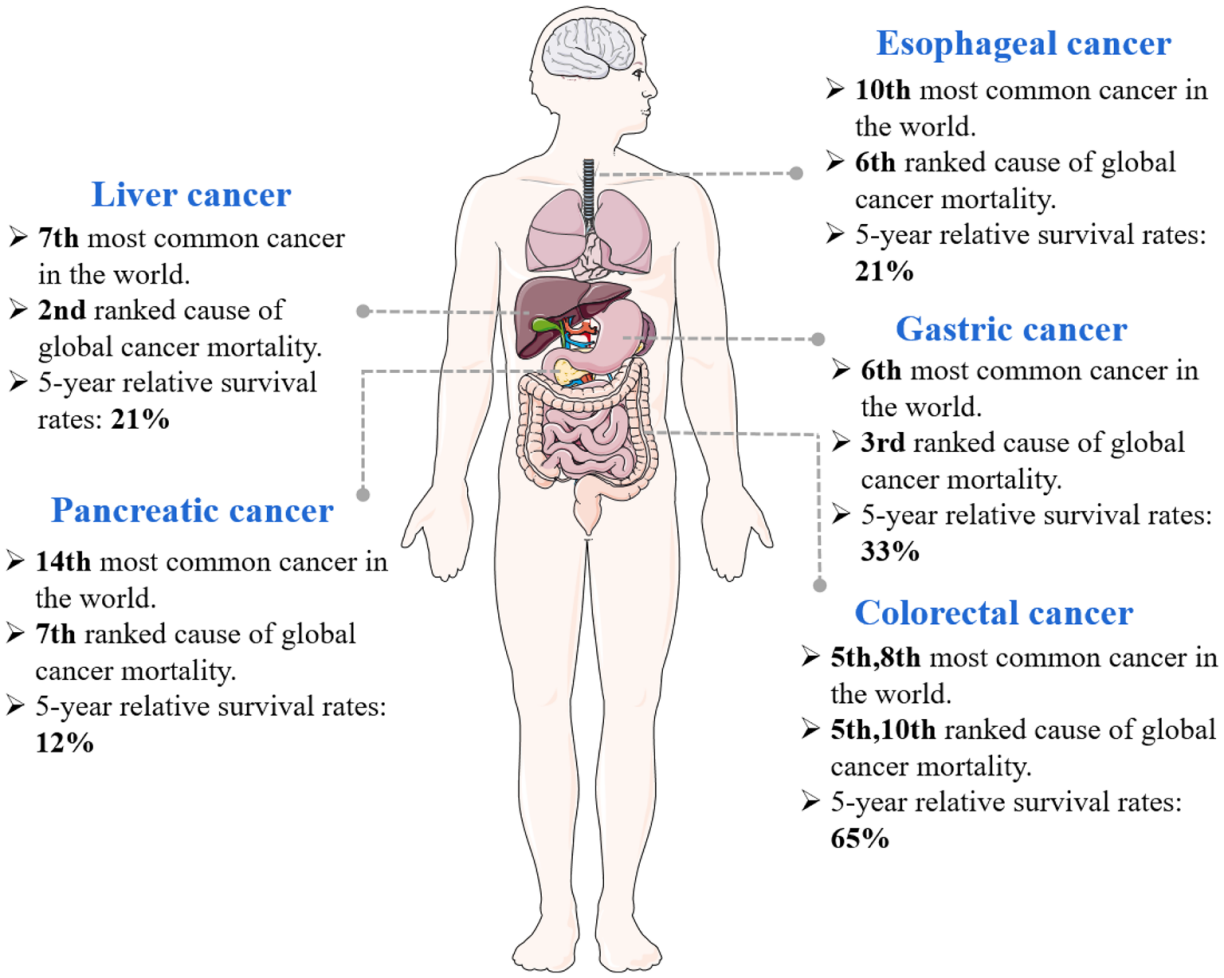
The distribution of GI cancer, their ranking among the top 10 most common cancer types in the world and causes of global cancer mortality, as well as 5-year relative survival rates

Gastrointestinal (GI) cancer is a kind of benign and malignant tumor originating in the human digestive tract, including gastric cancer (GC), esophageal cancer (EC), colorectal cancer (CRC), pancreatic cancer, liver cancer, etc. In China, GI cancer accounts for 45% of all cancer-related deaths except lung cancer [1]. Liver cancer increased from the third highest cancer mortality rate in 2018 to the second highest in 2020 [1]. Hepatocellular carcinoma (HCC) and cholangiocarcinoma (CCA) are the main subtypes of liver cancer, HCC accounts for 85% to 90%. Surgery remains the preferred treatment for liver cancer. Furthermore, targeted therapy, immunotherapy, liquid biopsy, and robot-assisted surgery are gradually applied to the clinical treatment of liver cancer [2]. GC is the sixth most common cancer and the third leading cause of cancer-related mortality. The majority (about 90%) of GC are adenocarcinomas, which occur in the superficial gland or mucosa of the stomach. Surgical treatments are commonly used for early and non-early stage operable GC, and cisplatin chemotherapy is used for advanced GC [3]. CRC is the fifth most common cancer in the world, with the tenth death rate among all cancers. Treatment modalities include local therapy (surgery, radiotherapy, ablative interventions) and systemic therapy (chemotherapy, targeted therapy, immunotherapy) [4]. Pancreatic cancer has low incidence among all cancers, but is still the seventh leading cause of cancer death. More than 90% of pancreatic cancers are pancreatic ductal adenocarcinoma (PDAC). Other types include acinocarcinoma, adensquamous carcinoma, and neuroendocrine tumors. Surgical resection is the only effective way for pancreatic cancer patients to obtain the chance of cure and long-term survival, other means such as radiotherapy, chemotherapy, interventional therapy, and optimal supportive therapy are also important ways to delay the disease of pancreatic cancer [5]. EC is the tenth most common cancer and the sixth leading cause of cancer death in the world, mainly including squamous cell carcinoma, adenocarcinoma, small cell carcinoma, with the first one comprising the majority of cases. Surgical treatment is one of the main radical methods for EC, and comprehensive means such as radiotherapy, systemic drug therapy, and endoscopic therapy are usually adopted after surgery [6]. The overview of GI cancer was shown in Fig. 1. Although traditional personalized cancer therapy has made progress, there are still a considerable number of patients with distant metastasis and drug resistance. Therefore, it is an urgent scientific problem to actively explore effective therapeutic targets and search for efficient targeted drugs for GI cancer.

Drug development based on natural products has always been an important direction of anti-tumor drug discovery and research. *Ganoderma lucidum (G. lucidum)* is a safe, non-toxic and versatile natural medicine that has been used for more than 2400 years. According to the 2020 edition of the Pharmacopoeia of the People's Republic of China, *G. lucidum* is the dried fruition body of *Ganoderma lucidum (Leyss. ex Fr.) Karst.* or *Ganoderma sinense Zhao, Xu et Zhang,* a species of fungus in the poraceae family. In modern medicine, *G. lucidum* has been used for preventing and treating different diseases, such as asthma [7], fatty liver [8], Alzheimer's disease (AD) [9], sleep disorder [10], and cancer [11]. Undeniably, the anti-tumor activity of *G. lucidum* has attracted high attention from researchers. Basic and preclinical studies have revealed that *G. lucidum*, alone or combined with drugs, can inhibit tumor cell proliferation, induce tumor cell apoptosis [12], inhibit tumor cell metastasis [13], regulate tumor cell autophagy [14], and so on. Clinically, the anti-tumor activity of *G. lucidum* has been verified in lung cancer [15], CRC [16], breast cancer [17], and other tumors. In traditional medicine, the role of *G. lucidum* in reinforcing the healthy *Qi* and eliminating the pathogenic factors is reflected in re-mobilizing the body's own repair ability, improving the internal environment, and enabling the body to achieve a state of *Yin* and *Yang* balance. For cancer patients with *Qi* deficiency and evil spirits abound, *G. lucidum* can be considered for simultaneous treatment of the symptoms and root cause, reinforce insufficiency and reduce excessiveness, effectively reduce cancer symptoms, and improve the survival rate of cancer patients, which is also in line with tumor pathogenesis type of modern medicine.

Therefore, based on existing research literature, the review discussed the role and mechanism of *G. lucidum* in GI cancer from four aspects from the perspective of Chinese and western medicine: first, the basic characteristics of *G. lucidum*; second, advances in anti-GI cancer effects of *G. lucidum;* third, clinical application of *G. lucidum*; fourth, the prospect of *G. lucidum*, so as to provide ideas and theoretical basis for further development and clinical application of *G. lucidum* in GI cancer.

## The basic characteristics of *G. lucidum*

*G. lucidum* is sweet and flat in taste, return to the heart, lung, liver and kidney meridians. It is registered in the Chinese pharmacopoeia for the efficacy of invigorating *Qi*, tranquilizing the mind, and relieving cough and asthma. It is an example of ancient remedy and known as immortality mushroom, and is widely distributed throughout the world, especially in China, Japan, and Korea [18, 19]. Sheng Nong’s herbal classic, the earliest pharmaceutical monograph in China, listed *G. lucidum* as the top quality and described the efficacy, which has the function of dissipate binds, and divided into green, red, yellow, white, black, purple according to the color of *G. lucidum*. In addition, the Compendium of Materia Medica, widely considered to be the most comprehensive medical literature in the history of traditional medicine in China, clearly pointed out that *G. lucidum* has the effect of invigorating spleen-stomach and replenishing *Qi*, protecting the liver, and so on, it is widely used in digestive tract diseases. These effects of *G. lucidum* were cited as a classic by subsequent generations of medicinists and have continued to this day. So far, State Food and Drug Administration of China (https://www.nmpa.gov.cn/datasearch/search-result.html) has approved a variety of *G. lucidum*-based drugs for clinical use, such as *G. lucidum* capsules and tablets, which have the effect of calming the heart, tranquilizing the mind, and strengthening the spleen and stomach; Shuganning injection, which contains the active ingredient of *G. lucidum*, has a good effect of reducing enzyme, relieving jaundice, and anti-inflammation in the treatment of viral hepatitis and other liver diseases; “G. sinense polysaccharide tablets” are approved as adjoint therapeutic agents for leukopenia and hematopoietic injury caused by chemotherapy/radiotherapy during cancer treatment in 2010 [20, 21].

**Fig. 2:**
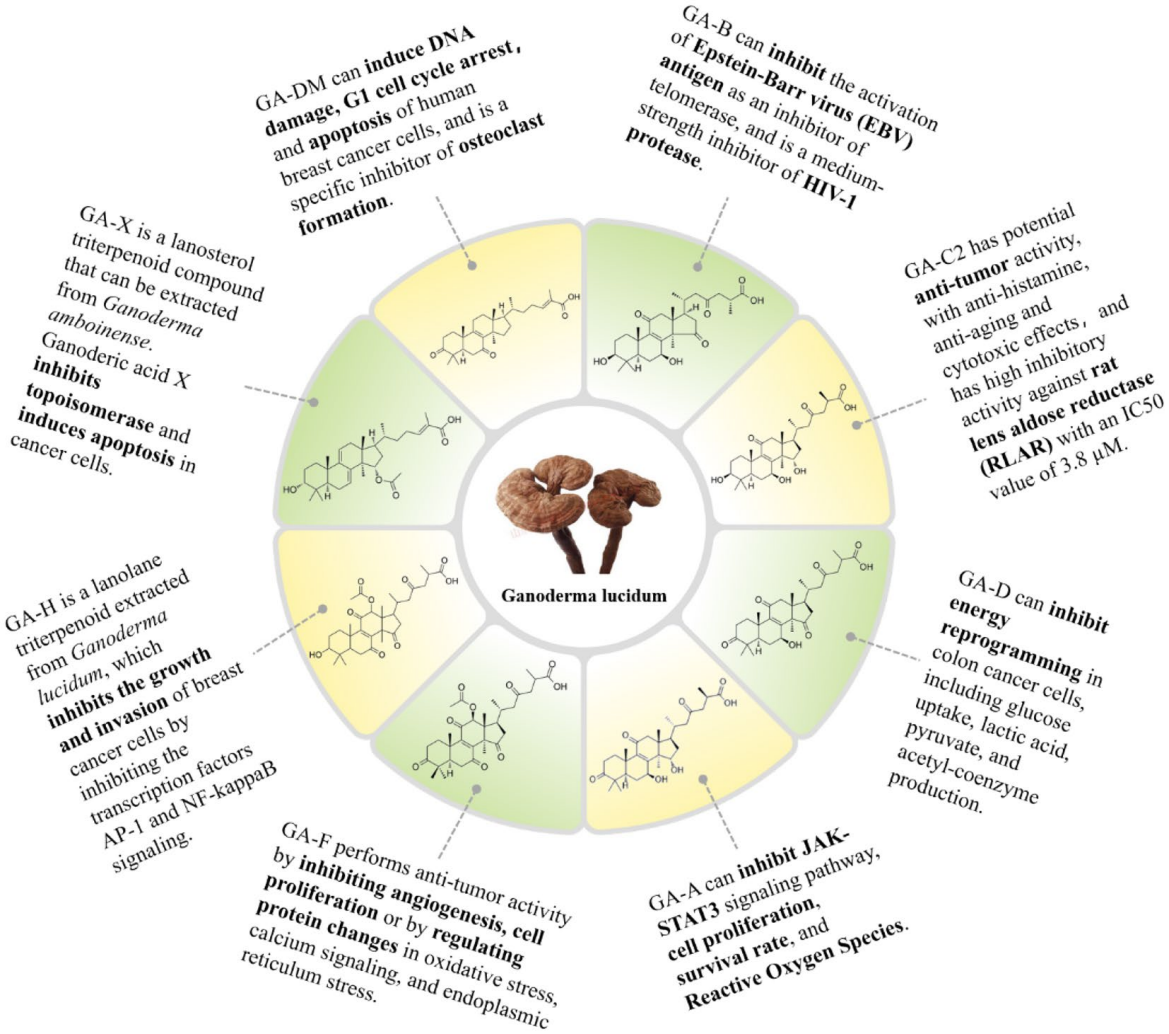
Structural formula of common GA found in *G. lucidum*. The figure shows the chemical structural formula of Ganoderic acid A, Ganoderic acid F, Ganoderic acid H, Ganoderic acid X, Ganoderic acid DM, Ganoderic acid B, Ganoderic acid C2, Ganoderic acid D

*G. lucidum* contains a variety of bioactive compounds, such as triterpenoids, polysaccharides, proteins, enzymes, vitamins, amino acids, flavonoids, steroids, alkaloids, and minerals [19, 22]. Triterpenoids and polysaccharides of *G. lucidum* are under the major consideration of studies due to their substantial pharmacological features. *G. lucidum* triterpenoids (GLT), a secondary metabolites of *G. lucidum*, is a highly oxidized lanostane derivatives [23], which mainly includes ganoderic acids (GA), lucidenic acids (LA), ganoderiol, ganodermantriol, lucialdehyde, and lanostanoid. In particular, GA has captured widespread attention due to its significant anti-tumor activity. Structural formula of common GA showed in Fig. 2. The extraction methods of GLT include traditional extraction methods such as organic solvent extraction, ultrasonic extraction, and enzymolysis extraction, as well as modern extraction methods such as supercritical fluid extraction, and high-voltage pulsed electric field extraction [24]. Most GLT have bitter taste, and the stronger the bitter taste, the higher the GLT content. *G. lucidum* polysaccharides (GLP) is a multi-carbohydrate molecule consisting of long chains of at least ten monosaccharide units linked together by glycosidic bonds, α-D-glucans, *β*-D-glucans, and polysaccharide-protein complex are the main active ingredients [25]. Among the separation methods of GLP, hot water extraction is the commonest, followed by methanol or ethanol precipitation. In addition, ultrasonic, microwave, and enzymatic methods are also used [26]. The difference in main chain and side chain structure of GLP resulted in the diversity in physiological activity. The longer main chain structure, the larger biomass, the higher biological activity.

Herein, the anti-tumor properties of the bioactive compounds and extracts of *G. lucidum* through inhibiting proliferation, inducing apoptosis, inhibiting metastasis, and regulating autophagy has been recombined in Tables 1, 2, 3, 4 and 5 for reference based on the research data published in the past 20 years (Tables 1, 2, 3, 4 and 5).

**Table 1:** The anti-cancer properties of some typical GLT compounds

GLT	Cancer	Anti-cancer mechanisms				Refs.
		Inhibiting proliferation	Inducing apoptosis	Inhibiting metastasis	Regulating autophagy	
GA	Hepatoma	** + **	−	−	−	[27]
GA-A	HCC	** + **	** + **	** + **	−	[28, 29]
GA-A	Neuroblastoma	** + **	−	−	−	[30]
GA-A	Glioblastoma	** + **	** + **	−	** + **	[31]
GA-A	Lung cancer	** + **	−	** + **	−	[32]
GA-A	Pancreatic cancer	** + **	−	−	−	[33]
GA-A	Cancer	** + **	** + **	−	−	[34]
GA-A	Brain glioma	** + **	−	−	−	[35]
GA-A	Prostate cancer	** + **	** + **	−	−	[36, 37]
GA-D	ESCC	** + **	** + **	−	** + **	[38]
GA-D	CRC	−	** + **	−	−	[39]
GA-DM	Breast cancer	** + **	** + **	−	−	[40]
GA-Me	CRC	** + **	** + **	−	−	[41]
GA-Me	Lung cancer	−	** + **	** + **	−	[42–44]
GA-T	Lung cancer	** + **	** + **	−	−	[45, 46]
GA-T	Colon cancer	** + **	−	** + **	−	[47, 48]
GA-T	Cervical cancer	** + **	** + **	−	−	[49]
GA-A/DM	Meningioma	** + **	** + **	−	−	[50]
GA-A/H	Breast cancer	** + **	-	** + **	−	[51]
GA-T/Mk	Cervical cancer	−	** + **	−	−	[52]
GA-Mf/S	Cervical carcinoma	−	** + **	−	−	[53]
LA-B	Hepatoma	−	−	** + **	−	[54]
Lucidumol D	Cancer	** + **	−	−	−	[55]
GAEE	Breast cancer	−	−	** + **	−	[56]

*ESCC* Esophageal squamous cell carcinoma; *GAEE G. lucidum* extract mainly contains *GA* dihydrogenated GA and GA isomer

+ : have the properties; -: lack of this properties

**Table 2:** The anti-cancer properties of some typical GLP compounds

GLP	Cancer	Anti-cancer mechanisms				Refs.
		Inhibiting proliferation	Inducing apoptosis	Inhibiting metastasis	Regulating autophagy	
FYGL	Pancreatic cancer	−	** + **	−	** + **	[57]
WSG	Tongue cancer	−	** + **	−	−	[58]
WSG	Lung cancer	−	−	** + **	−	[13]
SeGLP-2B-1	Breast cancer	** + **	** + **	−	−	[59]
(1,3)-*β*-D-Glucan derivative	Lymphoma	** + **	−	−	−	[60]
LZP-F3	Leukemia	−	** + **	−	−	[61]
LZP-F3	Urothelial carcinoma	−	** + **	−	−	[62]
SCGLP1	Osteosarcoma	** + **	** + **	−	−	[63]
GL-IV-I	Sarcoma	** + **	** + **	−	−	[64]
Ganoderan B	NSCLC	−	** + **	** + **	−	[65]
Ganopoly	Cancer	** + **	** + **	−	−	[66]
SeGLP-2B-1	Cancer	** + **	−	−	−	[67]
GLP-1–1	Cancer	** + **	−	−	−	[68]
*β*-glucan	Colon cancer	** + **	−	−	−	[69]

*FYGL* Fudan-Yueyang-*G. lucidum*; *LZP-F3* Ling-zhi polysaccharide fraction 3; *SCGLP1* Sulfated polysaccharide; *NSCLC* Non-small cell lung cancer; *GL-PP G. lucidum* peptide

+ : have the properties; -: lack of this properties

**Table 3:** The anti-tumor properties of GLP with different extraction methods

Extract solution	Cancer	Anti-cancer mechanisms				Refs.
		Inhibiting proliferation	Inducing apoptosis	Inhibiting metastasis	Regulating autophagy	
Hot water	CRC	** + **	** + **	−	** + **	[70, 71]
Hot water	Cervical carcinoma	−	** + **	−	−	[72]
Hot water	GC	−	** + **	−	** + **	[14]
Hot water	Colon cancer	−	** + **	** + **	−	[73]
Hot water	Prostate cancer	−	** + **	−	−	[74]
Enzymatic hydrolysate	CRC	−	** + **	−	−	[75]
Enzymatic hydrolysate	Cervical carcinoma	−	** + **	−	−	[76]

+ : have the properties; -: lack of this properties

**Table 4:** The anti-cancer properties of proteins isolated from *G. lucidum*

Other compounds	Cancer	Anti-cancer mechanisms				Refs.
		Inhibiting proliferation	Inducing apoptosis	Inhibiting metastasis	Regulating autophagy	
GLR	CRC	** + **	** + **	** + **	−	[77]
LZ-8	HCC	−	−	** + **	−	[78]
rLZ-8	GC	−	** + **	−	** + **	[79]
rLZ-8	Lung cancer	** + **	** + **	** + **	−	[80–82]
LZ-8,GMI	Lung cancer	−	** + **	** + **	−	[83]

*GLR G. lucidum* ribonuclease; *rLZ-8* recombinant Ling Zhi-8

+ : have the properties; -: lack of this properties

**Table 5:** The anti-cancer properties of other compounds isolated from *G. lucidum*

Other compounds	Cancer	Anti-cancer mechanisms				Refs.
		Inhibiting proliferation	Inducing apoptosis	Inhibiting metastasis	Regulating autophagy	
Ergosta-7,22-diene-2*β*,3α,9α-triol	Leukemia	−	** + **	−	−	[84]
9,11-dehydroergosterol peroxide	Melanoma	−	** + **	−	−	[85]
LingZhi oligopeptide-3	Lung cancer	−	** + **	−	−	[86]
5α, 8α-epidioxiergosta-6, 22-dien-3*β*-ol	HCC	−	** + **	** + **	−	[87]
GL-PP	Lung cancer	** + **	** + **	−	−	[88]
GL-PP	HCC	−	** + **	** + **	−	[89]

+ : have the properties; -: lack of this properties

At present, the research of *G. lucidum* tends to be a single active ingredient. Studies have shown that GLP could inhibit obesity, hyperlipidemia, inflammation, and fat accumulation in C57BL/6 J mice induced by high fat diet (HFD), and the mechanism is related to the up-regulation of toll-like receptor 4 (TLR4)/myeloid differentiation factor 88 (MyD88)/noncanonical nuclear factor-κB (NF-κB) signaling pathway [90]. GLT alleviated cognitive impairment and reduced the number of nerve fiber tangles in APP/PS1 transgenic AD model mice by inhibiting apoptosis and inactivating the rho-associated coiled-coil kinase (ROCK) signaling pathway. In vitro experiments, GLT promoted the proliferation of hippocampal neurons and had anti-oxidant effects [9]. GA-A, as one of the most abundant triterpenoids in *G. lucidum*, might improve alcoholic liver injury by regulating intestinal flora composition (elevating the content of Aerococcus, Bilophila, and Bifidobacterium) and liver metabolism spectrum, as well as mRNA levels of genes related to lipid metabolism and inflammatory response in the liver [91]. In addition, the bioactive compounds of *G. lucidum*, whether used alone or along with drugs, have proven to be effective in the prevention and treatment of multiple diseases. Li et al. demonstrated anti-aging effects of a *G. lucidum* preparation containing triterpenes and polysaccharides. It might improve testicular structure and function in middle-aged male mice by reducing oxidative stress, maintaining mitochondrial homeostasis, and inhibiting cell apoptosis [92]. El-Khashab et al. revealed that Atorvastatin and *G. lucidum* might have anti-tumor, pro-apoptosis, and anti-angiogenic activities by suppressing tumor growth in Ehrlich solid tumor. Notably, the combination of the two drugs improved anti-tumor activity [93]. Yuan et al. showed that the compound preparation of *G. lucidum* and *Rhodiola Rosea* could significantly alleviate cognitive impairment, ameliorate oxidative stress response, produce the immune enhancing effect, and decrease the secretion of inflammatory factors in aging model rats induced by D-galactose. The possible mechanism was to block the NF-κB signaling pathway by decreasing the MyD88 protein content in rats [94]. All up, *G. lucidum* cooperate with other drugs to produce a wide range of pharmacological effects.

## Advances in anti-GI cancer effects of *G. lucidum*

**Fig. 3:**
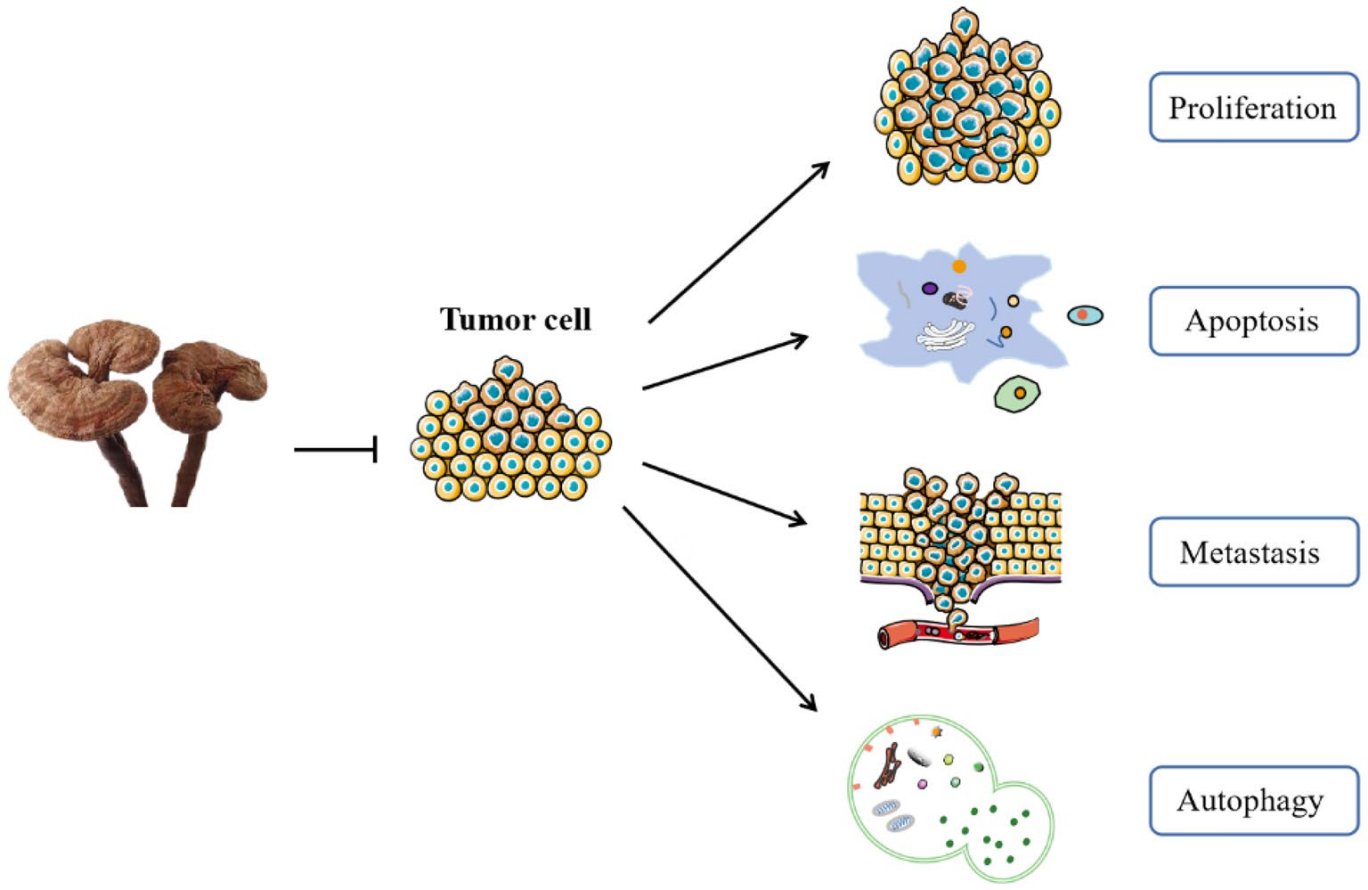
Mechanism of action of *G. lucidum* in the treatment of GI cancer: *G. lucidum* exerts anti-tumor activity mainly through inhibiting proliferation, inducing apoptosis, inhibiting metastasis, and regulating autophagy

GI cancer is the most frequent cancer in the world and one of the main causes of cancer-related death. *G. lucidum* is a widely used natural product with homology of medicine and food, it has advantages of less adverse reactions and multi-target regulation, and is often used in the treatment of GI cancer. In order to elaborate the effect of *G. lucidum* on GI cancer, the study collected data from article published in the past 20 years by referring to fourteen markers of cancer [95]. *G. lucidum* exerts anti-tumor activity mainly through inhibiting proliferation, inducing apoptosis, inhibiting metastasis, and regulating autophagy (Fig. 3, Table 6).

**Table 6:** Information on *G. lucidum* for the treatment of GI cancer

Cancer	*G. lucidum*	Cell type	Effects	Target/Mechanisms	Refs.
ESCC	*G. lucidum* spore powder	KYSE140 KYSE510	Anti-proliferation, anti-metastasis	PI3K/AKT/mTOR, Erk signaling pathways	[96]
ESCC	GA-D	EC9706 Eca109	Anti-proliferation, pro-apoptosis, pro-autophagy	mTOR signal transduction pathway	[38]
GC	GLP	MKN28 NCI-N87 AGS	Anti-proliferation, pro-apoptosis	Bcl-2/pro-caspase-3 cleaved-PARP/LC3-II/p62	[14]
GC	Cotreatment of GLE and QCT	SNU719 MKN1-EBV	Pro-apoptosis, anti-viral	Bcl-2/caspase 3/CYCS/EBV	[97]
GC	MGL	AGS	Anti-cancer	LC3-II/p62	[98]
GC	MGL	AGS	Anti-autophagy	LC3-II	[99]
GC	EGL	AGS	Pro-apoptosis	AKT signal pathway	[100]
GC	EGL	AGS	Anti-invasion	MMP-2/MMP-9	[101]
CCA	Supercritical-CO_2_ extract of *G. lucidum* spores	TFK-1	Anti-migration	Epithelial-mesenchymal transition	[102]
Hepatoma	Supercritical fluid extract of *G. lucidum*	BEL-7402	Anti-proliferation, pro-apoptosis	Blocking cell cycle	[103]
Hepatoma	GA	BEL7402	Anti-proliferation	Blocking cell cycle	[27]
HCC	GLP, ginsenoside Rg3, and oridonin	Huh7 HepG2	Pro-immune regulation, anti-proliferation	p-EGFR and AKT/GSK3 signaling pathways	[104]
HCC	GLP	SK-HEP-1 Huh-7	Anti-proliferation, anti-migration	PI3K/AKT signaling pathway	[105]
HCC	GLSP	SW1116	Pro-apoptosis	Regulating the PI3K/AKT signaling pathway	[106]
HCC	GL-PP	Huh7	Anti-migration, pro-apoptosis	No data	[107]
HCC	GLP	HepG2	Anti-proliferation, anti-migration	VEGF	[108]
HCC	GA-A	HepG2 SMMC7721	Anti-metastasis	Cyclin D1/p21/cleaved caspase 3	[29]
HCC	LAB	HepG2	Anti-invasion	ERK1/2/AP-1/ NF-κB /MMP-9	[54]
HCC	GLE	HepG2	Anti-proliferation, anti-metastasis	ERK1/2/c-Jun/c-Fos/AKT/MMP-2/MMP-9	[109]
Colon cancer	AGA	SW620 SW480 HT29	Anti-proliferation, anti-migration	P21/p53-dependent and independent manner	[110]
Colon cancer	EGLP	HCT116	Pro-apoptosis	Bax/p-ERK/cleaved caspase-3/Bcl-2/p-AKT1/COX-2	[75]
Colon cancer	GLT	HT-29	Pro-autophagy	Beclin-1/LC-3/p38 MAPK	[111]
CRC	WGL	HCT116	Anti-proliferation, pro-apoptosis	Cyclin A/B1/p21/p27	[70]
CRC	GLE	HCT116	Anti-proliferation, pro-apoptosis, pro-autophagy	Bcl-2/Bax/cleaved caspase-3/PARP	[112]
CRC	GA-Me	HCT116	Anti-proliferation, pro-apoptosis	AKT signal pathway	[41]
CRC	GLP	HCT116	Anti-proliferation, anti-migration, pro-apoptosis	caspase-8/Fas/caspase-3	[73]
CRC	EGL	HCT116	Anti-migration	E-cadherin/MMP-1/ MMP-2	[113]
CRC	GLP	LoVo	Anti-migration, pro-apoptosis	Fas/caspase-3/PARP	[114]
CRC	GLP	HT-29 HCT116	Pro-apoptosis	MAPK/ERK signaling pathway	[71]
CRC	GLR	HT29 HCT116	Anti-proliferative, anti-colony formation	ERK1/2/AP-1/NF-κB /MMP-9	[77]

*ESCC* Esophageal squamous cell carcinoma; *GLE G. lucidum* fruiting body; *QCT* Quercetin; GLSP, *G. lucidum* spore polysaccharide; *EGLP G. lucidum* polysaccharide enzymatic hydrolysate; *MGL* methanolic extract of *G. lucidum*; *GLR G. lucidum* ribonuclease; *EGL* ethanol extract of *G. lucidum*; *WGL* water extracts of *G. lucidum*

### The role of *G. lucidum* in cell proliferation

Cell proliferation, a vital component of cell growth and differentiation, is also an important life feature of organisms. However, in cancer, abnormal cell proliferation is a key link to promote its development [115]. Therefore, inhibiting the proliferation of cells is an effective way to combat cancer.

WGL inhibited HCT116 CRC cell proliferation induced by G2/M phase cell aggregation, possibly by down-regulating cyclin A and B1 and up-regulating p21 and p27. Studies on tumorigenesis in nude mice showed that WGL caused tumor shrinkage [70]. Additionally, Liu et al. found that GLE induced apoptosis, autophagy, and G0/G1 phase cell cycle arrest, hence inhibiting cell proliferation in HCT116 cells [112]. As such, *G. lucidum* might be a prospective, reliable therapeutic method for CRC. In another study, *G. lucidum* spore powder maigh inhibit the proliferation, migration, and invasion of ESCC cells via phosphoinositide 3-kinase (PI3K)/protein kinase B (AKT)/mammalian target of Rapamycin (mTOR) and extracellular signal-regulated kinase (ERK) pathway [96]. The supercritical fluid extract of *G. lucidum* (total component, TC), its acidic component (AC) and neutral component (NC) showed anti-hepatoma activity. NC or TC inhibited cell growth by arresting cell cycle in G2/M phase, while AC inhibited cell growth by preventing the transform from G1 to S phase [103]. GA, produced by submerged culture of *G. lucidum*, at 500 mg/mL, caused nearly a 70% inhibition of the growth of human hepatoma cell line BEL7402 but not of a normal human liver cell line L02 [27]. GA-Me, a pure lanostane triterpene isolated from *G. lucidum*, suppressed proliferation, caused DNA fragmentation, and markedly activated caspase-9 and caspase-3 in HCT116 cells. Moreover, whole-transcriptome sequencing and bioinformatics studies were carried out on HCT116 cells with or without GA-Me, the results suggested that GA-Me was a new multi-target compound with extensive pharmacological effects and molecular mechanisms. This study provided a new perspective for in-depth analysis of GA-Me [41].

GLP combined with ginsenoside Rg3, and oridonin could simultaneously target multiple signaling pathways to effectively inhibit HCC progression, including regulating immune function, reducing angiogenesis, and retarding proliferation [104]. AGA is a combination of traditional Chinese medicine *Antler’s* extract (A), *G. lucidum* (G), and *Antrodia camphorata* (A). Peng et al. demonstrated that AGA extract had potential to inhibit the proliferation, metastasis by inducing apoptosis in colon cancer. The underlying mechanism of these effects could be mediated through p53-independent/independent pathway. It is expected that AGA extract is a novel herbal anti-cancer drug for the treatment of colon cancer [110].

### The role of *G. lucidum* in cell apoptosis

Apoptosis is a spontaneous programmed death process in the body, which is a routine physiological phenomenon in multicellular organisms [116]. Currently, there are two main pathways of cell apoptosis: exogenous (death receptor induced apoptosis) signaling pathway, endogenous (mitochondria mediated apoptosis) signaling pathway. In addition, endoplasmic reticulum (ER) is also involved in cell apoptosis [117].

Caspases and B-cell lymphoma-2 (Bcl-2) are the key parts of cell apoptosis [118, 119]. EGLP might activate apoptosis in HCT-116 cell via up-regulation the expression of Bcl-2 associated X protein (Bax), phospho-ERK (p-ERK), and cleaved caspase-3, down-regulation the expression of Bcl-2, phospho-serine/threonine kinase 1 (p-AKT1), and cyclooxygenase-2 (COX-2) [75]. GLP inhibited the growth and metastasis of HCT 116 cells by up-regulating the expression of caspase-8, fatty acid synthase (Fas), and caspase-3 through intracellular calcium release and death receptor pathways [73]. Jang et al. demonstrated that EGL triggered apoptosis through activation of the intrinsic caspase pathway along with the death receptor (DR)-mediated extrinsic pathway, thereby inhibiting the growth of AGS cells [100]. Abnormal activation of PI3K/AKT signaling pathway can promote the proliferation and inhibit apoptosis of cancer cells [120]. Shen et al. demonstrated that GLP could repress the proliferation and migration of SK-HEP-1 and Huh-7 cells by regulating PI3K/AKT signaling pathway, and induce G1 cell cycle arrest and apoptosis [105]. Similarly, GLSP could alter macrophage polarity and induce apoptosis of hepatocellular carcinoma cells by triggering PI3K/AKT signaling pathway [106]. The Ras/Raf/mitogen-activated protein kinases (MAPK)/extracellular signal-regulated kinase (MEK)/ERK signaling pathway is one of the vital regulatory pathways of tumor cell apoptosis. Zhu et al. found that G85, a triterpenoid-rich extract with high-pressure supercritical CO_2_ from *G. lucidum*, could significantly inhibit the proliferation and induce apoptosis of liver cancer cells via suppression of Ras/Raf/MEK/ERK signaling pathway [121]. Moreover, Zhong et al. demonstrated that GLP could induce apoptosis of human GC cells by interfering with autophagy flux, and confirmed that *G. lucidum* spore powder had strong potential to inhibit cancer cell proliferation in MKN28, NCI, N87, and AGS cell [14]. Further, the author explored the molecular mechanism of *G. lucidum* against GC based on the molecular docking technology of network pharmacology and cell experiments, and the results showed that *G. lucidum* played a synergistic role against GC through multi-component, multi-target, and multi-signal channels, among which apoptosis was undoubtedly the most important signaling pathway [122].

Cotreatment of *G. lucidum* extract and quercetin at low concentration synergistically reduced cell viability and induced apoptosis, showing anti-tumor and anti-viral activity against SNU719 epstein-barr virus (EBV)-associated GC cells. Remarkably, the addition of GA-A could produce biological activity similar to that of *G. lucidum* extract [97].

### The role of *G. lucidum* in cell metastasis

Cell metastasis plays an indispensable role in the occurrence and progression of cancer. Malignant tumor metastasis includes cytoskeletal remodeling [123], epithelial-mesenchymal transformation [124], enhanced migration and invasion ability [125], microenvironment change [126], immune escape [126], and other processes, which is one of the leading causes of death in cancer patients, and an important factor affecting the prognosis of patients. Therefore, inhibiting the metastasis of cancer cells is a prospective therapeutic strategy for cancer.

Matrix metalloproteinases (MMPs), including the gelatinases MMP-2 and MMP-9, are a family of secreted or transmembrane proteins that can degrade the proteins of the extracellular matrix (ECM). MMPs have been implicated in many abnormal physiological conditions, including cancer invasion, and metastasis [47]. Recent research has documented that EGL significantly inhibited the formation and growth of xenografts in nude mice and the migration of HCT116 cell. This effect was related to the significant up-regulation of E-cadherin and the down-regulation of MMP-1 and MMP-2 [113]. Previously published research documented that GLE significantly suppressed the number of metastatic tumor-bearing mice, the number of affected organs, and the number of tumor foci as well as the MMP-2 and -9 activities in serum of mice [109]. In vitro, Weng et al. demonstrated that the anti-invasion effect of the LAB on the phorbol-12-myristate-13-acetate (PMA)-induced HepG2 cells might be through inhibiting the phosphorylation of ERK1/2 and reducing activating protein-1 (AP-1) and NF-κB DNA-binding activities, leading to down-regulation of MMP-9 expression [54]. Likely, MMPs serve as the crucial target of EGL-induced anti-invasiveness in AGS cells, which could inhibit mRNA and protein expression of MMP-2 and MMP-9 in a dose-dependent manner [101]. Further analysis demonstrated that a supercritical-CO_2_ extract of *G. lucidum* spores suppressed the transforming growth factor beta1 (TGF-β1)-induced migration of TFK-1 via inhibition of epithelial-mesenchymal transition (EMT) [102]. Besides, it was observed in the LoVo cell scratch experiment that migration was significantly inhibited after incubation with 0.625–10 mg/mL GLP, which may be related to up-regulation of Fas and caspase-3 protein expression and down-regulation of poly (ADP-ribose) polymerase (PARP) protein expression [114], GLP could also obstruct the migration of HepG2 by down-regulating vascular endothelial growth factor (VEGF) protein expression [108]. Huang et al. discovered that GL-PP could significantly inhibit the migration of HCC (Huh7). Following the raise of GL-PP concentration, the migration inhibition becomes more and more obvious, showing a dose–effect relationship. However, the specific mechanism of GL-PP inhibiting Huh7 migration remains to be further studied [107]. In another study, HepG2 and SMMC7721 human HCC cell lines were treated with GA-A at different concentrations for 24, 48, and 72 h. Transwell experiment was adopted to test cell migration and invasion, and the results revealed that, compared with the control group, the number of cells migrated to the lower chamber through the membrane and the number of invasive cells were observably reduced in the GA-A treatment group (*P* < 0.01) [29].

### The role of *G. lucidum* in cell autophagy

Autophagy is an essential process to maintain the stability of the intracellular environment. Under the regulation of autophagy-related genes, defective organelles and macromolecules are eliminated by lysosomes [127]. Autophagy plays a double-edged role in tumors, which can enhance or block the survival of tumors according to the stages of tumors and different tumor tissues [128]. This suggests that regulating autophagy can be an effective intervention strategy for cancer treatment.

GLR is a protein isolated from *G. lucidum* that inhibits CRC activity. Dan et al. reported that it inhibited the autophagy activation of HT29 and HCT116 cells, with accumulation of P62, up-regulation of light chain 3-I (LC3-I), and down-regulation of LC3-II [77]. Also, the effect of GA-D on ESCC cells has revealed that it could activate autophagy and promote the autophagosomes formation, along with blocking the fusion of autophagy and lysosome to trigger autophagy cell death [38]. Reis et al. observed that the MGL promoted the formation of autophagosomes (typical autophagic vacuoles) in human GC cells, and the expression of p62 and LC3-II proteins was increased when cells was stimulated with MGL and lysosomal protease inhibitors compared with MGL alone. These results confirmed that *G. lucidum* extract was an autophagy inducer [98]. Actually, some researchers previously reported that MGL has the effect of interfering autophagy and cell cycle on the growth of AGS cell [99]. Pan et al. carried out some interesting experiments to demonstrate that GLP could induce autophagy and apoptosis of CRC HT-29 and HCT116 cells by activating MAPK/ERK pathway. In vivo, GLP could inhibit the growth and autophagy flux of tumor cells. These results suggested that GLP could be used as an autophagy initiation inducer and also as an innovative autophagic flux inhibitor through blocking autophagosome-lysosome fusion [71]. Thyagarajan et al. showed that GLT suppressed growth of HT-29 cells through cell cycle arrest at the G0/G1 phase and by the induction of the programmed cell death Type II, autophagy. Moreover, GLT also inhibited growth of tumors in a xenograft model of colon cancer [111].

## Clinical application of *G. lucidum*

Clinically, anti-tumor effect of *G. lucidum* is achieved primarily through enhancing the immune system. There are a few clinical trials which have been conducted on exclusively *G. lucidum*, while most of clinical studies have been done in combination therapy with chemotherapy, radiotherapy, and other drugs for cancer treatment [129–131]. Moreover, multi-drug resistance (MDR) is a major obstacle for successful tumor therapy, leading to the generation of insensitive cancer cells towards administered therapy [132]. Accordingly, the role of *G. lucidum* in reversing MDR has also aroused the interest of some researchers, and relevant clinical studies are gradually being carried out.

### The role of *G. lucidum* in immunotherapy

Tumor immunotherapy is the fourth method of tumor treatment after surgical resection, chemotherapy, and radiotherapy [133]. It mainly activates the patient's own immune system, and enhances their anti-tumor immunity, thereby controlling and killing tumor cells. It is considered to be the only method that has the potential to completely eliminate tumor cells, and is the most promising treatment method in the comprehensive treatment of tumors.

The increase of regulatory T cells (Treg cells) in peripheral blood and tumor has been shown to be related to the worse prognosis of HCC patients [134]. As a result, the amount and function of targeted Treg cells has been a target for HCC therapy. GLP could markedly suppress tumor growth in hepatoma-bearing mice, and the percentage of Treg cells in tumors reduced in a dose-dependent manner. Furthermore, inactivation of tumor-infiltrating Treg cells could abolish the anti-tumor activity of GLP [135]. These results indicated that GLP directly suppressed the growth of liver tumors by reducing the accumulation and activation of Treg cells. Tumor necrosis factor α (TNF-α) is a great hallmark to activate cellular immunity, interleukin 1beta (IL-1β) and interleukin 6 (IL-6) are significant pro-inflammatory factors excreted from M1 macrophages [136, 137]. Xia et al. reported that GLP might accelerate the secretion of TNF-α, IL-6, and IL-1β through inducing CD68 macrophages, decrease the inhibitory effect of interleukin 13 (IL-13) secreted by natural killer T lymphocyte on tumor immune monitoring, enhance the immune function of organism, so that the growth of distal tumors in HCC mice is inhibited [138]. Song et al. demonstrated that GLSP stimulated macrophages to restructure the tumor microenvironment, accelerated the polarization of primary macrophages to M1 type, and promoted the secretion of TNF-α, IL-1β, IL-6, TGF-β1, and other inflammatory factors and cytokines [106]. Additionally, GLP could alleviate the occurrence of colitis and tumor in AOM/DSS induced mice. Compared with the control group, CD68 and F4/80 (macrophage surface markers) were obviously elevated in AOM/DSS induced mice. In order to further investigate the role of GLP on the function of immune cells, a cell model was established in vitro, the results indicated that GLP could suppress the activation and inflammation of RAW264.7 macrophages induced by lipopolysaccharides (LPS), possibly regulated by TLR4/MyD88/NF-κB, and MAPK inhibition [139]. Sliva et al. studied the effects of GLT on mouse model of colon cancer induced by foodborne carcinogen and inflammation, and found that GLT could suppress colon tumor formation, reduce focal hyperplasia, and the number of aberrant crypt foci. In addition, GLT also had certain inhibitory effect on inflammation and could reduce the infiltration of macrophages in colon [140].

### The role of *G. lucidum* in reversing multidrug resistance

MDR means that after a tumor cell becomes resistant to a certain type of chemotherapy drug, it will also develop cross-resistance to a variety of other chemotherapy drugs, which is the leading cause of chemotherapy failure and tumor recurrence in cancer patients [141, 142]. Therefore, overcoming MDR is the key to continuously improve the clinical effect of tumor chemotherapy and finally treat malignant tumors.

ABCB1, a 170 kDa transmembrane glycoprotein encoded by MDR1 gene, is widely distributed in MDR cancer cells. ABCB1 has abundant chemotherapeutic substrates, including vinblastine, doxorubicin, etoposide, paclitaxel, and bisantrene [143]. GA-B greatly heightened the susceptibility of HepG2/ADM to doxorubicin, vinblastine, paclitaxel and other ABCB1 substrates. Moreover, GA-B did not change the susceptibility of HepG2/ADM cells to cisplatin, a non-ABCB1 substrate, suggesting that the reversal effect of GA-B was associated with ABCB1-induced drug resistance [144]. GA-A played an important role in improving the chemical sensitivity of HepG2 cells to cisplatin and accelerated cisplatin induced cell death via inhibiting the Janus kinase (JAK) signal transductor as well as transcription activator 3 (STAT3) signaling pathway, thereby promoting cisplatin induced cell death. These observations suggested that the combination of GA-A and chemotherapy drugs for cancer therapy was a potential therapeutic strategy [145]. GA-A also strengthened the tumor inhibitory effect of oxaliplatin on xenograft model, but has no significant effect on the proliferation and apoptosis of HT-29 cells stimulated by oxaliplatin, and a single dose of GA-A had no obvious anti-tumor effect. Meaningfully, the combination of oxaliplatin and GA-A had no significant effect on the T lymphocyte subtypes of xenotransplantation. T lymphocyte toxicity was significantly increased in co-administered mice compared with oxaliplatin treated mice. These data suggested that GA-A might synergistically enhance the inhibitory effect of oxaliplatin on tumors by elevating cytotoxicity of T cells [146]. GA-Me significantly enhanced the cytotoxicity of vincristine, oxaliplatin induced HCT-8/VCR and HCT-116/l-OHP cells, and effectively reversed the multidrug resistance of MDR colon cancer cells by suppressing the function of hMDR1 promoter, the expression level of MRPs, and adjusting apoptosis-related pathways [147]. GLP could reverse tumor MDR. The reversal mechanism might be: increased drug accumulation, decreased drug efflux, or increased intracellular drug concentration by reducing the expression level of MDR1 gene, and affecting P-glycoprotein expression. GLP has very low toxicity and good reverse effect, so it may become a low-toxicity and efficient reverse agent for tumor MDR [148].

### Clinical efficacy of *G. lucidum* in cancer

Preclinical studies have demonstrated powerful anti-tumor effects of *G. lucidum*. Again, there are some clinical evidence to support this. Increased cytokines (such as TNF-α and IL-1) are believed to hastened cancer cachexia, with the symptoms of weight loss, anorexia, tiredness, and anemia [149]. Medicines that down-regulates TNF-α and IL-1 have been shown to improve cancer cachexia [150]. Research suggested that *G. lucidum* had potential immunomodulatory effects in patients with advanced CRC. After 12 weeks of treatment with *G. lucidum*, the expression levels of TNF-α and IL-1 in 73.2% of the patients studied were decreased. Therefore, *G. lucidum* might be an effective way to improve cancer cachexia [129]. *G. lucidum* spore capsule combined with chemotherapy played a satisfactory treatment effect on GI cancer, such as GC, esophageal cancer, liver cancer, CRC, etc., and significantly improves patients' immune function and quality of life [130]. Ganopoly, an extract of GLP, could stimulate host defense response by enhancing the activity of NK cell and promoting the secretion of IL-2 and, interferon-γ (TFN-γ), thus enhancing immune regulatory function in advanced cancer patients [151]. Deng et al. discussed the effect of *G. lucidum* spore powder intervention during adjuvant chemotherapy on postoperative immune system of patients with breast cancer and lung cancer, and the research found that T cell activation was strongly related to inflammatory cytokines, AGR, NLR, and *G. lucidum* therapy [17]. Sun et al. detected peripheral blood of lung cancer patients and found that plant hemagglutinin was abnormally activated in the plasma, and lymphocyte proliferation, CD69 expression, perforin, and granulozyme B production were inhibited, while GLP could partially or completely reverse this effect [15]. A water-soluble extract from culture medium of *G. lucidum* mycelia (MAK), one of the extracts from *G. lucidum*. After taking MAK (1.5 g/ day) for 12 months, 52% of patients had at least one reduction in adenoma, the amount and total size of adenomas were obviously reduced from baseline [16]. The data indicated that MAK inhibited the progression of colorectal adenomas-precancerous lesions of the large bowel. Zhuang et al. demonstrated that administration of the aherb complex (CCMH; a mixture of *citronellol* and extracts of *G. lucidum*, *C. pilosula* and *A. sinensis*) for 6 weeks significantly elevated the number of immune cells and reduced the number of leukopenia and neutropenia, as well as NK cell and CD4 lymphocyte in cancer patients in process of chemotherapy and/or radiotherapy [131]. The *Reishi* & *Privet* formula (RPF) is made up of dried sporederm-broken spores of the artificially cultivated *G. lucidum* and ethanol extracts, water extracts from the dried mature fruit of *Ligustrum lucidum*. Liu et al. preliminarily proved the safety of RPF, and showed that RPF had a bright prospect in maintaining the living quality and emotional health of patients with NSCLC chemotherapy [152].

## Conclusion and future prospect

GI cancer are a major disease threatening human life and health. *G. lucidum*, a traditional natural product, has unparalleled advantages in the treatment of GI cancer due to its wide range of pharmacological activities and application pathways. This review summarizes the basic characteristics of *G. lucidum*, the anti-tumor properties of its compounds and extracts, and the research progress of *G. lucidum* in the treatment of GI cancer in the past 20 years, focusing on the anti-GI cancer mechanism and clinical application.

Current studies have confirmed that pharmacologically active compounds and extracts of *G. lucidum* have clear anti-GI cancer effects. Among them, GA, such as GA-A, GA-B, GA-Me, and GA-D, have shown outstanding advantages in anti-GI cancer, especially in reversing MDR, and is expected to be a potential leader of anti-tumor drugs. In addition, several studies showed that *G. lucidum* might exhibit synergistic effects or superior anti-tumor activities in combination with other agents/drugs, which could be an attractive alternative in the future clinical study of *G. lucidum* [93, 97, 153].

However, there are still some problems: first, although the research on the anti-tumor effect of *G. lucidum* has reached the molecular level, its direct target and specific molecular mechanism are still unclear, and more in-depth research is needed; second, the researches on the pharmacological action of *G. lucidum* are mostly confined to basic studies such as cell, animals, and few clinical studies have reported. Therefore, further clinical trials and evidence-based medicine are required to assess the safety and effectiveness of *G. lucidum* in treating human cancer; third, the trend of combination therapy of *G. lucidum* is not mature enough, and the relevant research data needs to be further improved. The combination of *G. lucidum* with chemotherapy, radiotherapy, and other drugs may be an effective way to achieve complementary advantages, reduce toxicity, increase efficiency, and thus control tumor cell proliferation and metastasis. In conclusion, this review offers a comprehensive overview of the current advancements in the research on *G. lucidum*, while also contemplating future directions in exploring its potential anti-tumor effects. This serves to provide a valuable reference for subsequent research pertaining to *G. lucidum*. Despite the fact that existing research on *G. lucidum* has not yet reached the point of constituting a principal clinical method for anti-tumor treatment, it holds potential. The unceasing, in-depth exploration of cancer pathogenesis and the properties of *G. lucidum* may well pave the way for its potential application in anti-tumor therapy. This continuous research contributes significantly to the broader sphere of anti-tumor studies, particularly those involving natural pharmaceutical ingredients, including *G. lucidum*.

## Data Availability

Not applicable.
